# Markers of gut mucosal inflammation and cow’s milk specific immunoglobulins in non-IgE cow’s milk allergy

**DOI:** 10.1186/2045-7022-4-8

**Published:** 2014-03-05

**Authors:** Laura Merras-Salmio, Kaija-Leena Kolho, Anna S Pelkonen, Mikael Kuitunen, Mika J Mäkelä, Erkki Savilahti

**Affiliations:** 1 Children’s Hospital, Division of Pediatric Gastroenterology, Helsinki University Central Hospital, PO BOX 281, FIN-00029 HUS, Helsinki, Finland; 2 Department of Pediatric Allergology, Helsinki University Central Hospital, Helsinki, Finland; 3 Children’s Hospital, Helsinki University Central Hospital, Helsinki, Finland

## Abstract

**Background:**

Allergy to cow’s milk protein (CMP) may cause gastrointestinal (GI) symptoms in the absence of CMP specific IgE. The immunological mechanisms involved in such disease are not fully understood. Therefore we examined markers of gut mucosal inflammation and the immunoglobulin profiles in children with Gl symptoms suspected of cow’s milk protein allergy (CMPA).

**Patients and methods:**

We prospectively recruited infants and young children (n = 57; median age 8.7 months) with gastrointestinal complaints suspected of CMPA. The diagnosis of CMPA was made using the double-blind, placebo-controlled food challenge. Serum and stool samples were collected during CMP-free diet and after both placebo and active challenges. We analyzed the stool samples for calprotectin, human β-defensin 2 and IgA. In serum, we analyzed the levels of β-lactoglobulin and α-casein specific IgA, and IgG antibodies (total IgG and subclasses IgG1 and IgG4). Control group included children with e.g. dermatological or pulmonary problems, consuming normal diets.

**Results:**

Fecal calprotectin levels were higher in the challenge positive group (n = 18) than in the negative (n = 37), with respective geometric means 55 μg/g [95% confidence interval 38–81] and 29 [24–36] μg/g (p = 0.0039), during cow’s milk free diet. There were no significant inter-group differences in the fecal β-defensin and IgA levels. The CMP specific IgG and IgA were not elevated in patients with CMPA, but the levels of β-lactoglobulin-IgG4 (p = 0.0118) and α-casein-IgG4 (p = 0.0044), and total α-casein-IgG (p = 0.0054) and -IgA (p = 0.0050) in all patient samples (regardless of CMPA diagnosis) were significantly lower compared to the control group using dairy products.

**Conclusions:**

Despite cow’s milk elimination in children intolerant to cow’s milk there might be ongoing low-grade inflammation in the gut mucosa. CMP specific IgG or IgA should not be used to diagnose non-IgE CMPA. The observed frequency of impaired CMP specific total IgA, IgG and IgG4 production in patients following cow’s milk free diet warrants further studies.

## Introduction

Intolerance to cow’s milk protein may cause gastrointestinal (GI) symptoms in infants. Such symptoms (in the absence of atopic dermatitis) are usually non-IgE-mediated. In the food-protein-induced enterocolitis syndrome (FPIES) the suggested immunological (or allergic) pathophysiology mainly involves T-cell-mediated reactions, as recently reviewed [1]. Other immunological responses suggested in the pathogenesis of the GI symptoms associating with cow’s milk protein allergy (CMPA) include increased mononuclear cell production of tumor necrosis factor-α (TNF-α) [2], eosinophilic inflammation [3,4] as well as activated submucosal mast cells [5]. Possible non-immunological etiologies include 1) the direct effect of cow’s milk protein on intestinal motility [6], 2) colonic bacterial dysbiosis (possibly enhanced by cow’s milk formulas) [7,8], and 3) effects caused by the non-protein parts of cow’s milk: carbohydrates [9] and fatty acids [10].

When CMPA is manifested by slowly developing gastrointestinal symptoms it should be separated from the more common and easily-diagnosed IgE-mediated food allergy [11]. In gastrointestinally manifested CMPA (GI-CMPA), the only reliable method of diagnosis is the double-blind, placebo-controlled food challenge (DBPCFC), and cow’s milk specific IgE antibodies and skin prick tests (SPTs) are usually negative. The prognosis for the non-IgE food allergy is more favorable. Tolerance to cow’s milk in CMPA patients has been shown to develop after age one, with nearly 100% of the IgE-negative patients being tolerant by age three, compared to 60% of the IgE positive [12]. The levels of IgG4 subclass antibodies to CM specific allergens were lower in the DBPCFC positive than in the negative infants with eczema suspected of CMPA [13]. In GI-CMPA, school-age children (4.0-10.8 years, n = 14) suffering from non-IgE CMPA associated gastrointestinal complaints had increased levels of β-lactoglobulin-IgG4 compared to controls [14], whereas in adults patients (n = 19) with self-reported intolerance to CMP, the α-casein and β-lactoglobulin-IgG4 levels were similar to those in the controls [15]. CMP specific IgG4 data regarding infant patients with GI-CMPA is lacking.

Fecal calprotectin, a granulocyte protein binding calcium that exerts antimicrobial effects, is an applicable surrogate marker for gut mucosal inflammation [16], but it has not been studied in GI-CMPA patients. Increased calprotectin levels at six months of age have been associated with lower risk of later atopic sensitization [17]. Human β-defensins belong to a group of antimicrobial peptides expressed in the epithelial cells as part of the innate immunity. Among their pleiotropic functions β-defensins can activate antigen-presenting cells. Increased levels of fecal β-defensin 2 in six-month-old children may associate with later risk of atopic sensitization [18]. To better understand the pathology of symptoms interpreted as GI-CMPA, it is crucial to obtain more knowledge about what happens in the gut mucosa in children with symptoms suggestive of GI-CMPA.

The aims of the present study were 1) to examine markers of inflammation derived from the gut mucosa, and 2) to measure the total IgG, IgA, and IgG subclasses (especially IgG4) to cow’s milk specific proteins, in children suspected of having GI-CMPA.

## Methods

We prospectively recruited children between 0 and 4 years of age, who had been referred to the Pediatric Allergy Unit (a tertiary level hospital for allergic and skin diseases) on suspicion of gastrointestinally manifested CMPA during the year 2010. All of the participants (n = 57) were following a cow’s milk free diet and underwent DBPCFC for cow’s milk and skin prick tests (SPTs) for cow’s milk and hen’s egg. After a baseline evaluation we started the DBPCFC for cow’s milk using the same hypoallergenic formula as a placebo that the child had used during the CM elimination period (an extensively hydrolyzed formula, an amino acid –based formula or a soy formula). Parents kept a standard symptom diary, including items on daily stool consistency (Bristol score) and frequency, and possible infectious symptoms, one week before the challenges and during the challenge period. The details of our five-day DBPCFC protocol were recently published [11]. We took serum and stool samples prior to the DBPCFC, and during each provocation period (3–5 days after the start of either the active or placebo provocation). The fecal samples were sent to laboratory in room temperature and stored at −20°C.

The control group consisted of 22 children between the ages of 0 and 4, who were recruited during the study period when they visited the Pediatric Allergy Unit for other reasons than food allergy, atopic eczema, or atopic asthma, such as suspected drug allergies, and non-atopic wheezing.

The methodology used for the SPTs has been described in a previous study [11]. Reactions with a mean wheal diameter of 3 mm or larger were considered positive. We determined serum cow’s milk-specific IgE (Phadia Immunocap, detection limit 0.35 kU/l) using the routine, commercially available method.

### Measurement of CM and ovalbumin specific antibodies

Serum antibodies of IgA, IgG, IgG1, and IgG4 isotypes for cow’s milk β-lactoglobulin and α-casein and ovalbumin were measured using enzyme-linked immunosorbent assays (ELISA) with the results reported as Arbitrary Units (AU), based on previous reports for the antigen- specific IgA and IgG [19], and for the antigen-specific IgG4 and IgG1 [20]. The Arbitrary Units (AU) are deduced from the optical densities of the reference serum curve with a high level of antibodies after subtracting the blanks. The reference serum was a separate pool of sera collected for IgA and IgG isotypes; its concentration was assigned to 100 AU. The detection limits (expressed as AU) were as follows: α-casein-specific IgA 0.26; IgG 0.70; IgG1 0.28; IgG4 0.28, β-lactoglobulin-specific IgA 0.3; IgG 0.028; IgG1 0.028; IgG4 0.028, and ovalbumin-specific IgA 1.5; IgG 0.26; IgG1 1.3; IgG4 5.20.

### Measurement of fecal β-defensin 2, fecal IgA, and fecal calprotectin

Fecal calprotectin was analyzed immediately using PhiCal Test (Calpro AS, Oslo, Norway; NovaTec Immunodiagnostica, Dietzenbach, GmBH, Germany). Prior to fecal β-defensin 2 and Immunoglobulin A (F-IgA) analyses, thawed samples were homogenized in phosphate-saline buffer and centrifuged for 15 min at 10 000 g, +4°C, to retrieve a supernatant for the measurements. We applied enzyme linked immunosorbent assays (ELISA) according to the manufacturers’ instructions for measuring fecal levels of human β-defensin 2 (Immundiagnostik, Bensheim, Germany; detection limit 0.077 ng/mL). Fecal IgA was determined using ELISA, according to the method described before [21]. The detection limit for F-IgA was 5 μg IgA/l.

### Statistical analyses

We analyzed the laboratory data for the three different study groups (DBPCFC positive patients, DBPCFC negative patients, and control patients) using the non-parametric ANOVA (Kruskall-Wallis) test to compare three medians. One-to-one comparisons were done using the non-parametric Mann-Whitney’s test (if unpaired data) or in the case of paired data, using the Wilcoxon matched-pairs signed rank test. Spearman’s test was employed for determining the correlations. We report geometric means for each measurement, with 95% confidence intervals for this mean. Values below the detection limit (in the serum antibody samples only) were computed by dividing the detection limit by two. We conducted all of the analyses using GraphPad Prism software (version 5.0 for Mac) and considered p-values below 0.05 as significant.

### Ethics

This study was approved by the Helsinki University Hospital Ethics Committee (No.317/13/03/03/2009). Both legal guardians gave their written informed consent before the child was enrolled in the study.

## Results

The clinical characteristics of the patients suspected of having CMPA based on gastrointestinal symptoms and those of the control group are depicted in Table 1. The majority of patients presented with excessive crying or fussiness, vomiting or loose stools, with almost half of them reporting three or more symptoms. The DBPCFC ruled out cow’s milk protein intolerance for the majority: only 18/57 (32%, median age 8.4 months[2.4-40.8]) of the DBPCFC’s were deemed positive. At least one associated symptom was loose stools for the majority (14/18) of positive challenges, while the most common (in 77%) symptom among the DBPCFC negative patients (median age 8.7 months[2.5-25.6]) was excessive crying/fussiness. A more detailed report on the DBPCFC results can be found in a recently published article [11]. None of the patients or controls had detectable levels of cow’s milk specific IgE. Two patients in each study group had a wheal diameter of 3 mm for the CM SPT; one of the DBPCFC positive patients also had a positive SPT for hen’s egg (4 mm). All of these SPT reactions were within a low probability range for IgE-mediated allergic disease [22]. None of these children showed signs of such at the six-month follow-up.

**Table 1:** Characteristics of pediatric patients with gastrointestinal symptoms suggestive of non-IgE cow’s milk allergy

	**All patients**	**Challenge negative**	**Challenge positive**	p-value*	**Controls (with no food allergies)**
	**n = 57**	**n = 39**	**n = 18**		**n = 22**
Age, months (median, range)	8.7 (2.4–40.8)	8.7 (2.5 –25.6)	8.4 (2.4–40.8)	NS	13.2 (4.8–30)
Duration of CM free diet before challenge, months (median, range)	2.5 (0.5–35)	2.4 (0.5–14)	2.5 (0.5–35)	NS	NA
CM specifig IgE > 0.35 kU/l	0	0	0	NS	0
SPT wheal size ≥3 mm for cow’s milk	4	2	2	NS	ND
Symptoms associated with CMA suspicion					
Loose stools, n (%)		18 (46%)	14 (78%)	0.0432	
During CM challenge		1	15	0.0001	
Placebo challenge		10	1		
Excessive crying, n (%)		30 (77%)	11 (56%)	NS	
During CM challenge		2	8	NS	
Placebo challenge		15	4		
Vomiting/reflux, n (%)		19 (49%)	8 (44%)	NS	
During CM challenge		0	3	NS	
Placebo challenge		7	2		
Constipation, n (%)		10 (26%)	2 (11%)	NS	
During CM challenge		0	0	NS	
Placebo challenge		0	0		
Skin eruptions, n(%)		10 (26%)	5 (28%)	NS	
During CM challenge		2	2	NS	
Placebo challenge		2	3		

CM Cow’s Milk.

SPT Skin Prick test.

CMA Cow’s milk allergy.

NS Not significant.

NA Not applicable.

ND Not done.

*p-values below 0.05 are considered significant.

This table is modified from Merras-Salmio et al. [11].

**Figure 1:**
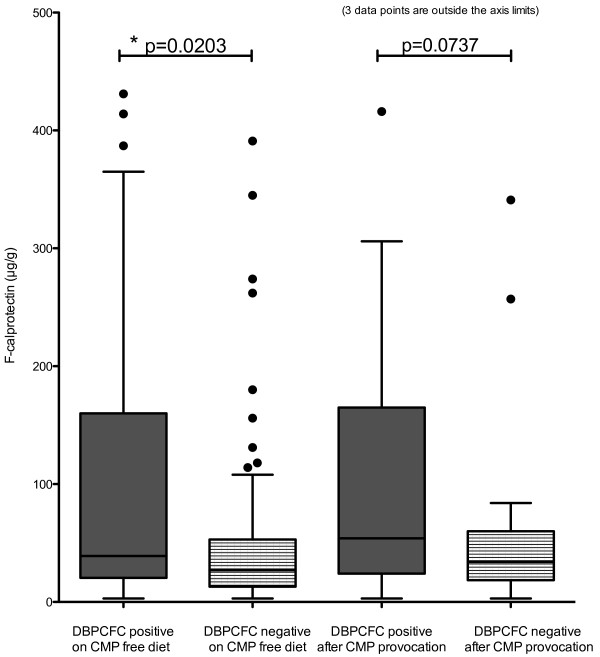
**Fecal calprotectin in children (n = 57) with suspicion of gastrointestinally manifested cow’s milk allergy.** The double-blind, placebo-controlled food challenge (DBPCFC) was used to diagnose the cow’s milk allergy: n = 18 for DBPCFC positive patients and n = 39 for DBPCFC negative patients. The graph shows Tukey’s whiskers with medians. (CMP = Cow’s Milk Protein).

Fecal calprotectin levels were higher in the DBPCFC positive group than in the challenge negative group: the respective geometric means being 55 μg/g [95% confidence Interval: 38–81] and 29 μg/g [24–36] (p = 0.0039). Table 2 and Figure 1 show the results of further subdivision into samples taken during cow’s milk free diet and after the challenge with cow’s milk. The within-group variation in calprotectin levels was high in both patient groups, compared to controls with a slightly higher median age. Fecal calprotectin correlated weakly with fecal IgA in the DBPCFC positive group (Spearman correlation coefficient 0.4583, p = 0.0567) and in the control group (Spearman correlation coefficient 0.5875, p = 0.0272), but not in the DBPCFC negative group. Fecal β-defensin 2 and IgA showed high levels of within-group variation and a trend towards higher median levels in the DBPCFC positive, but the differences between the groups were not significant (Table 2). Fecal occult blood test positivity (in two DBPCFC patients pre-challenge and in four DBPCFC negative patients either pre- or post challenge) did not associate with the levels of fecal inflammatory markers (data not shown).

**Table 2:** Fecal calprotectin, IgA and β-defensin 2 levels in young children (n = 57) with gastrointestinal symptoms suggestive of non-IgE cow’s milk allergy

		**Cow’s milk free diet**	**Cow’s milk challenge†**	**p-value**	**Controls (n = 22)**
Fecal calprotectin (μg/g)	CMA positive (n = 18)	52 [33–86]*	60 [30–122]	*0.5995*	25 [13–50]
	CMA negative (n = 39)	28 [21–36]*	33 [24–44]	*0.4674*	
p-value		*0.0203	*0.0737*		
Fecal IgA (g/l)	CMA positive (n = 18)	0.54 [0.37–0.79]	0.48 [0.36–0.62]	*0.4509*	0.33 [0.22–0.51]
	CMA negative (n = 39)	0.46 [0.35–0.61]	0.45 [0.36–0.55]	*0.3138*	
p-value		*0.6349*	*0.8086*		
Fecal β-defensin2 (ng/ml)	CMA positive (n = 18)	38.6 [19.6–75.7]	47.50 [23.1–97.8]	*0.1354*	20.8 [8.6–50.0]
	CMA negative (n = 39)	22.4 [14.5–34.6]	31.12 [20.5–47.3]	*0.1144*	
p-value		*0.1976*	*0.2520*		

Cow’s milk allergy was diagnosed with the double-blind, placebo-controlled food challenge. Values in table are geometric means [95% Confidence Interval of the mean].

*Mann–Whitney test p-values reported between the two patients groups. Values below 0.05 are considered significant.

† Fecal samples taken 3–5 days after start of cow’s milk containing challenge formula.

The α-casein-specific levels of IgG4 and total IgG and IgA, in comparison to the controls were lower in the study patients (Table 3). We found no significant differences between the DBPCFC positive patients and the DBPCFC negative patients. The levels of β-lactoglobulin IgG4 in the study patients were also independent of the DBPCFC result, and lower than those for the control group using cow’s milk normally (p = 0.0029, Table 3). The levels for β-lactoglobulin total IgG and IgG1, and α-casein IgG1 did not differ between the three groups. Ovalbumin-specific IgA, IgG, IgG1 and IgG4 levels were similarly low in all groups (Table 3). None of the patients had suspected or proven hen’s egg allergy either at baseline or during follow-up.

**Table 3:** Pre-challenge levels of serum α-casein, β-lactoglobulin and ovalbumin specific IgA, IgG (total) and IgG4 in young children with gastrointestinal symptoms suggestive of non-IgE cow’s milk allergy

	**DBPCFC negative**	**DBPCFC positive**	p-values*	**Controls(with no diet restrictions)**	p-values**
	**n = 39**	**n = 18**		**n = 22**	
**α-casein**					
Total IgG	1.16 (0.80–1.70)	1.36 (0.64–2.88)	0.8410	4.16 (2.01–8.60)	0.0054*
IgG1	0.54 (0.30–1.0)	0.52 (0.25–1.11)	0.7300	0.84 (0.31–2.26)	0.7761
IgG4	0.07 (0.05–0.10)	0.09 (0.04–0.19)	0.6394	0.21 (0.11–0.37)	0.0024*
Total IgA	1.46 (1.00–2.13)	1.33 (0–71–2.50)	0.4969	4.14 (2.20–7.80)	0.0050*
**β-lactoglobulin**					
Total IgG	0.43 (0.28–0.70)	0.27 (0.10–0.71)	0.3924	0.44 (0.27–0.69)	0.5770
IgG1	0.026 (0.009–0.07)	0.04 (0.01–0.19)	0.5463	0.05 (0.001–0.20)	0.6848
IgG4	0.004 (0.001–0.009)	0.005 (0.0001–0.03)	0.9362	0.06 (0.01–0.29)	0.0029*
Total IgA	0.35 (0.24–0.51)	0.35 (0.20–0.60)	0.9743	0.96 (0.50–1.86)	0.0100*
**Ovalbumin**					
Total IgG	2.67 (1.24–5.75)	3.40 (0.95–12.14)	0.5857	3.27 (0.95–11.27)	0.8053
IgG1	0.58 (0.27–1.25)	0.95 (0.28–3.24)	0.4520	1.26 (0.40–4.00)	0.3756
IgG4	0.11 (0.05–0.23)	0.25 (0.07–0.92)	0.1652	0.21 (0.05–0.73)	0.3299
Total IgA	1.34 (0.97–1.86)	0.89 (0.69–1.1)	0.1147	1.82 (0.95–3.48)	0.1087

These levels are expressed as Arbitrary Units (AU). Cow’s milk allergy was diagnosed with the double-blind, placebo-controlled food challenge (DBPCFC). Reported figures are geometric means (with 95% confidence interval of the mean).

*The Mann–Whitney test p-value between the two patient groups.

**p-values calculated by multiple comparison ANOVA (nonparametric Kruskall-Wallis test), comparing the two patient groups and the control group.

## Discussion

Gastrointestinally manifested (non-IgE) CMA is a diagnostically challenging disease. Despite our attempts, we did not find laboratory measurements that could serve as an additional tool for the diagnosis at the individual level. However, the average fecal calprotectin levels of the DBPCFC positive patients following a cow’s milk free diet were higher than those of the challenge-negative patients and controls. There was no further increase in the calprotectin levels after provocation with cow’s milk. The difference between the groups was small, and most values still remained within the normal range but this finding certainly raises the need for future studies. The levels of both β-lactoglobulin and α-casein specific IgG4; and α-casein total IgG and IgA, were significantly low in both patient groups on cow’s milk free diet compared to the controls without diet restrictions.

In infancy, calprotectin levels are known to vary. The median age of our patients was close to 9 months in both the challenge negative and positive groups. Low grade mucosal inflammation, as suggested by the increased calprotectin, may augment gut permeability and endorse the development of allergies in susceptible patients [23], but also contrary evidence exists [17]. The gut microbiota composition (higher values for clostridia and staphylococci colonization) affects the stool calprotectin excretion in neonates [24]. In infants with colicky crying, fecal calprotectin levels tend to be higher than in the non-crying controls [25], with aberrant colonic bacterial colonization [26]. On the other hand, at the age of three months, babies on CM formula had lower calprotectin levels than those who were breast-fed [27]. Regarding the here presented evidence suggesting low-grade gut mucosal inflammation continuing during the cow’s milk free diet, it is of interest that according to a registry study in Finland, there is a distinct association between the diagnosis of gastrointestinally manifested CMPA in early childhood and the later development of IBD in adolescence [28]. Unfortunately, the high variation in the calprotectin levels in the present study renders this test unfit for use in the diagnostic process for GI-CMPA, but this finding certainly needs to be further studied.

The low or undetectable levels of IgG and IgA antibodies to bovine α-casein and β-lactoglobulin deserve attention. In infancy, although a relative immaturity exists in producing antibodies IgG and IgA in total, especially IgG4 production is low with the nadir at 4–6 months of age and reaching adult levels only in adolescence [29]. Therefore the slightly different median ages in patients and controls may partly explain this finding. However, levels of IgG4 were only in the control group similar to those previously published in asymptomatic infants (median age six months), using the same method [13]. Levels of β-lactoglobulin-IgG4 in that study where lower in the CMPA patients with mucocutaneous symptoms (combining IgE and non-IgE-mediated reactions). Another study on older children with challenge-proven non-IgE GI-CMPA reported levels of β-lactoglobulin-IgG4 to be higher than in the controls [14]. In the present study, ovalbumin IgG4 levels were similar between the three groups, and in line with the previous work [13], indicating unperturbed capability to age-appropriate production of IgG4. Thus the low levels of CMP specific IgG4 (and α-casein specific total IgG and IgA) we found likely reflect the cow’s milk free diet in the patients, using whey hydrolysate formulas or total CM elimination. The non-allergic controls used CM normally. Higher or rising levels of specific IgG4 antibodies associate with developing tolerance to cow’s milk in IgE mediated CMPA [30,31]. It is unclear whether a prolonged CM free diet in a non-allergic infant could predispose to the development of CMPA. Worsening of IgE-mediated food allergy after elimination diets have been reported [32]. Our findings do indicate that the CM free diet associates with decreased production of most CM specific immunoglobulins. On the other hand, since higher levels of the CM specific IgGs or IgA were found in asymptomatic controls, these tests should not be used in diagnosing GI-CMPA.

Fecal IgA levels taken during cow’s milk elimination diet showed a trend towards higher values in the DBPCFC positive patients. A previous study on 25 patients suspected of non-IgE CMPA also failed to demonstrate a significant difference in fecal secretory IgA levels between the CMPA positive and negative [33]. A study pooling both IgE and non-IgE-mediated CMPA did find significantly higher fecal IgA levels in patients (pre-challenge) in those with positive cow’s milk challenge than in the challenge negative [21]. As none of our patients had CMP specific IgE, the fecal IgA secretion may not be as relevant in non-IgE CMPA.

This study has certain limitations. Though amongst the largest published for gastrointestinally manifesting non-IgE CMPA, the sample size was still relatively small, affecting in particular the DBPCFC positive group. To address the association between calprotectin and gut mucosal proinflammatory markers, larger studies on patients with a suspicion of gastrointestinally manifested, non-IgE mediated CMPA are needed. Also, due to sample size, separating the DBPCFC positive patients based on their symptoms or other clinical parameters was not feasible. None of our patients presented with symptoms suggestive of food-protein induced enterocolitis (immediate pallor, vomiting and subsequent diarrhea [34]), which has indeed become a rarity in our clinical practice. The stool samples that we collected in this study were not immediately frozen, but instead were transported at room temperature to laboratory within one to two days. This proved to be feasible when measuring fecal calprotectin [35], but the lengthy preservation of the samples at room temperature may have had a minor effect on fecal IgA and β-defensin.

The major strength of this study is that it is one of the largest studies published so far that focuses on the gastrointestinal manifestations of CMPA. The use of the DBPCFC in the present study adds to the credibility of our results. It is obvious from previous research that a non-IgE mediated food intolerance/allergy has a distinct pathophysiology compared to the IgE mediated allergies. The results from studies focusing on IgE mediated CMPA are probably not relevant in managing patients with non-IgE mediated GI-CMPA. The DBPCFC protocol we used was feasible in the daily clinical practice and well received by the parents. In fact, the use of a DBPCFC should be mandatory when diagnosing GI-CMPA, due to the frequently observed placebo symptoms [11]. Even with the DBPCFC, the likelihood of false positive diagnoses here is significant. We did not use repeated testing to overcome this possibility, as many GI-CMPA patients suffer from problematic feeding, and prolonging the other dietary restrictions (as required by the food challenge protocol) further would have been unethical.

In conclusion, we found evidence indicating low-grade inflammation in gut mucosa as suggested by slightly elevated fecal calprotectin levels in infants who tested positive for GI-CMPA (presenting with diarrhea and vomiting) during the cow’s milk challenge. The increase in calprotectin was independent of cow’s milk elimination or provocation. This finding may indicate a novel pathophysiology behind the symptoms associated with GI-CMPA, but fecal calprotectin cannot be used in the diagnostic process due to the high degree of variation in the levels. We also discovered that the dietary elimination of cow’s milk protein results in low levels of CM specific IgA, total IgG and IgG4. Whether this associates with slower development of tolerance to cow’s milk needs to be further studied. The findings address the importance of accurate diagnostics of GI-CMPA and emphasize that the need of strict cow’s milk elimination should be thoroughly considered.

## Abbreviations

CMP: Cow’ milk protein; DBPCFC: Double-blind, placebo-controlled food challenge; CMPA: Cow’s milk protein allergy; GI: Gastrointestinal; GI-CMPA: Gastrointestinally manifested cow’s milk protein allergy; FPIES: Food –protein induced enterocolitis syndrome; SPT: Skin prick test.

## Competing interests

The authors declare no conflicts of interest. This study has been financially supported by the Lahja and Väinö Kivi Foundation, the Päivikki and Sakari Sohlberg Foundation, a Helsinki University Hospital Research Grant, and National Graduate School of Clinical Investigation.

## Authors’ contributions

LM-S wrote the first draft of the article, was involved in the design and implementation of the study, and performed the statistical analyses. K-LK, ASP and MJM were involved in the study design and implementation, as well as in interpreting the results and writing of the article. ES was involved in the study design, laboratory testing and interpreting the final results as well as writing of the article. All authors read and approved the final manuscript.

## References

[B1] Caubet JC Nowak-Wegrzyn A Current understanding of the immune mechanisms of food protein-induced enterocolitis syndrome Expert Rev Clin Immunol 2011 7 317 327 10.1586/eci.11.13 21595598

[B2] Benlounes N Candalh C Matarazzo P Dupont C Heyman M The time-course of milk antigen-induced TNF-alpha secretion differs according to the clinical symptoms in children with cow’s milk allergy J Allergy Clin Immunol 1999 104 863 869 10.1016/S0091-6749(99)70300-3 10518834

[B3] Chang JW Wu TC Wang KS Huang IF Huang B Yu IT Colon mucosal pathology in infants under three months of age with diarrhea disorders J Pediatr Gastroenterol Nutr 2002 35 387 390 10.1097/00005176-200209000-00031 12352535

[B4] Arvola T Ruuska T Keranen J Hyoty H Salminen S Isolauri E Rectal bleeding in infancy: clinical, allergological, and microbiological examination Pediatrics 2006 117 e760 e768 10.1542/peds.2005-1069 16585287

[B5] Schappi MG Borrelli O Knafelz D Williams S Smith VV Milla PJ Lindley KJ Mast cell-nerve interactions in children with functional dyspepsia J Pediatr Gastroenterol Nutr 2008 47 472 480 10.1097/MPG.0b013e318186008e 18852640

[B6] Vandenplas Y Veereman-Wauters G De Greef E Devreker T Hauser B Benninga M Heymans HS Gastrointestinal manifestation of cow’s milk protein allergy or intolerance and gastrointestinal motility J Pediatr Gastroenterol Nutr 2011 53 Suppl 2 S15 S17 22235453

[B7] Francavilla R Calasso M Calace L Siragusa S Ndagijimana M Vernocchi P Brunetti L Mancino G Tedeschi G Guerzoni E Indrio F Laghi L Miniello VL Gobbetti M De Angelis M Effect of lactose on gut microbiota and metabolome of infants with cow’s milk allergy Pediatr Allergy Immunol 2012 23 420 427 10.1111/j.1399-3038.2012.01286.x 22435727

[B8] Thompson-Chagoyan OC Fallani M Maldonado J Vieites JM Khanna S Edwards C Dore J Gil A Faecal microbiota and short-chain fatty acid levels in faeces from infants with cow’s milk protein allergy Int Arch Allergy Immunol 2011 156 325 332 10.1159/000323893 21720179

[B9] Moore DJ Robb TA Davidson GP Breath hydrogen response to milk containing lactose in colicky and noncolicky infants J Pediatr 1988 113 979 984 10.1016/S0022-3476(88)80567-5 3193321

[B10] Kanwar RK Macgibbon AK Black PN Kanwar JR Rowan A Vale M Krissansen GW Bovine milk fat enriched in conjugated linoleic and vaccenic acids attenuates allergic airway disease in mice Clin Exp Allergy 2008 38 208 218 18005183 10.1111/j.1365-2222.2007.02868.x

[B11] Merras-Salmio L Pelkonen A Kuitunen M Kolho K Mäkelä M Cow’s milk associated symptoms evaluated using the double-blind, placebo-controlled food challenge J Pediatr Gastroenterol Nutr 2013 57 3 281 286 10.1097/MPG.0b013e3182993fe0 23974059

[B12] Saarinen KM Pelkonen AS Makela MJ Savilahti E Clinical course and prognosis of cow’s milk allergy are dependent on milk-specific IgE status J Allergy Clin Immunol 2005 116 869 875 10.1016/j.jaci.2005.06.018 16210063

[B13] Savilahti EM Viljanen M Kuitunen M Savilahti E Cow’s milk and ovalbumin-specific IgG and IgA in children with eczema: low beta-lactoglobulin-specific IgG4 levels are associated with cow’s milk allergy Pediatr Allergy Immunol 2012 23 590 596 10.1111/j.1399-3038.2012.01277.x 22435658

[B14] Sletten GB Halvorsen R Egaas E Halstensen TS Changes in humoral responses to beta-lactoglobulin in tolerant patients suggest a particular role for IgG4 in delayed, non-IgE-mediated cow’s milk allergy Pediatr Allergy Immunol 2006 17 435 443 10.1111/j.1399-3038.2006.00408.x 16925689

[B15] Hochwallner H Schulmeister U Swoboda I Twaroch TE Vogelsang H Kazemi-Shirazi L Kundi M Balic N Quirce S Rumpold H Froschl R Horak F Tichatschek B Stefanescu CL Szepfalusi Z Papadopoulos NG Mari A Ebner C Pauli G Valenta R Spitzauer S Patients suffering from non-IgE-mediated cow’s milk protein intolerance cannot be diagnosed based on IgG subclass or IgA responses to milk allergens Allergy 2011 66 1201 1207 10.1111/j.1398-9995.2011.02635.x 21575008

[B16] Gisbert JP McNicholl AG Questions and answers on the role of faecal calprotectin as a biological marker in inflammatory bowel disease Dig Liver Dis 2009 41 56 66 10.1016/j.dld.2008.05.008 18602356

[B17] Kukkonen K Kuitunen M Haahtela T Korpela R Poussa T Savilahti E High intestinal IgA associates with reduced risk of IgE-associated allergic diseases Pediatr Allergy Immunol 2010 21 67 73 10.1111/j.1399-3038.2009.00907.x 19566584

[B18] Savilahti EM Kukkonen AK Haahtela T Tuure T Kuitunen M Savilahti E Intestinal defensin secretion in infancy is associated with the emergence of sensitization and atopic dermatitis Clin Exp Allergy 2012 42 405 411 10.1111/j.1365-2222.2011.03904.x 22093109

[B19] Savilahti E Saukkonen TT Virtala ET Tuomilehto J Akerblom HK Increased levels of cow’s milk and beta-lactoglobulin antibodies in young children with newly diagnosed IDDM. The Childhood Diabetes in Finland Study Group Diabetes Care 1993 16 984 989 10.2337/diacare.16.7.984 7993386

[B20] Savilahti EM Saarinen KM Savilahti E Duration of clinical reactivity in cow’s milk allergy is associated with levels of specific immunoglobulin G4 and immunoglobulin A antibodies to beta-lactoglobulin Clin Exp Allergy 2010 40 251 256 10.1111/j.1365-2222.2009.03409.x 19958365

[B21] Saarinen KM Sarnesto A Savilahti E Markers of inflammation in the feces of infants with cow’s milk allergy Pediatr Allergy Immunol 2002 13 188 194 10.1034/j.1399-3038.2002.01027.x 12144641

[B22] Verstege A Mehl A Rolinck-Werninghaus C Staden U Nocon M Beyer K Niggemann B The predictive value of the skin prick test weal size for the outcome of oral food challenges Clin Exp Allergy 2005 35 1220 1226 10.1111/j.1365-2222.2005.2324.x 16164451

[B23] Perrier C Corthesy B Gut permeability and food allergies Clin Exp Allergy 2011 41 20 28 10.1111/j.1365-2222.2010.03639.x 21070397

[B24] Rouge C Butel MJ Piloquet H Ferraris L Legrand A Vodovar M Voyer M de la Cochetiere MF Darmaun D Roze JC Fecal calprotectin excretion in preterm infants during the neonatal period PLoS One 2010 5 e11083 10.1371/journal.pone.0011083 20552029 PMC2884033

[B25] Rhoads JM Fatheree NY Norori J Liu Y Lucke JF Tyson JE Ferris MJ Altered fecal microflora and increased fecal calprotectin in infants with colic J Pediatr 2009 155 823 828 e821 10.1016/j.jpeds.2009.05.012 19628216

[B26] de Weerth C Fuentes S Puylaert P de Vos WM Intestinal microbiota of infants with colic: development and specific signatures Pediatrics 2013 131 e550 e558 10.1542/peds.2012-1449 23319531

[B27] Savino F Castagno E Calabrese R Viola S Oggero R Miniero R High faecal calprotectin levels in healthy, exclusively breast-fed infants Neonatology 2010 97 299 304 10.1159/000255161 19887860

[B28] Virta LJ Ashorn M Kolho KL Cow’s milk allergy, asthma and pediatric inflammatory bowel disease J Pediatr Gastroenterol Nutr 2013 56 649 651 10.1097/MPG.0b013e318285e9d8 23319082

[B29] Zegers BJ van der Giessen M Reerink-Brongers EE Stoop JW The serum IgG subclass levels in healthy infants of 13–62 weeks of age Clin Chim Acta 1980 101 265 269 10.1016/0009-8981(80)90252-1 7357748

[B30] Ito K Futamura M Moverare R Tanaka A Kawabe T Sakamoto T Borres MP The usefulness of casein-specific IgE and IgG4 antibodies in cow’s milk allergic children Clin Mol Allergy 2012 10 1 10.1186/1476-7961-10-1 22212305 PMC3398319

[B31] Savilahti EM Rantanen V Lin JS Karinen S Saarinen KM Goldis M Makela MJ Hautaniemi S Savilahti E Sampson HA Early recovery from cow’s milk allergy is associated with decreasing IgE and increasing IgG4 binding to cow’s milk epitopes J Allergy Clin Immunol 2010 125 1315 1321 e1319 10.1016/j.jaci.2010.03.025 20462631 PMC3289532

[B32] Barbi E Gerarduzzi T Longo G Ventura A Fatal allergy as a possible consequence of long-term elimination diet Allergy 2004 59 668 669 15147454 10.1111/j.1398-9995.2004.00398.x

[B33] Kalach N Kapel N Waligora-Dupriet AJ Castelain MC Cousin MO Sauvage C Ba F Nicolis I Campeotto F Butel MJ Dupont C Intestinal permeability and fecal eosinophil-derived neurotoxin are the best diagnosis tools for digestive non-IgE-mediated cow’s milk allergy in toddlers Clin Chem Lab Med 2013 51 351 361 23087088 10.1515/cclm-2012-0083

[B34] Katz Y Goldberg MR Rajuan N Cohen A Leshno M The prevalence and natural course of food protein-induced enterocolitis syndrome to cow’s milk: a large-scale, prospective population-based study J Allergy Clin Immunol 2011 127 647 653 e641-643 10.1016/j.jaci.2010.12.1105 21377033

[B35] Roseth AG Fagerhol MK Aadland E Schjonsby H Assessment of the neutrophil dominating protein calprotectin in feces. A methodologic study Scand J Gastroenterol 1992 27 793 798 10.3109/00365529209011186 1411288

